# Effect of arginine vasopressin on systemic and pulmonary arterial pressure in a patient with pulmonary hypertension secondary to pulmonary emphysema: a case report

**DOI:** 10.1186/s40981-016-0072-3

**Published:** 2017-01-04

**Authors:** Toshiyuki Mizota, Kohei Fujiwara, Miho Hamada, Shino Matsukawa, Hajime Segawa

**Affiliations:** Department of Anesthesia, Kyoto University Hospital, 54 Shogoin-Kawahara-Cho, Sakyo-Ku, Kyoto, 606-8507 Japan

**Keywords:** Pulmonary hypertension, Hypotension, Vasopressor, Arginine vasopressin, Noradrenaline

## Abstract

Although data from several studies support the use of arginine vasopressin (AVP) for the treatment of hypotension concomitant with pulmonary hypertension (PH) in the cardiac surgery setting, to our knowledge, no previous studies have reported the effect of AVP on the systemic and pulmonary circulation of patients with PH secondary to lung diseases. In this report, we present the hemodynamic responses to bolus administrations of AVP and noradrenaline in a patient with PH secondary to pulmonary emphysema. The patient showed low systemic vascular resistance hypotension during off-pump single-lung transplantation. The bolus administration of AVP (0.5 U) increased systemic arterial pressure by 35.2%, with a minimal change in pulmonary arterial pressure, resulting in a significant decrease in the pulmonary arterial pressure/systemic arterial pressure ratio. In contrast, the bolus administration of noradrenaline (10 or 20 μg) increased both systemic and pulmonary arterial pressures by 14.8 and 6.7%, respectively. In summary, the bolus administration of AVP effectively increased systemic arterial pressure with a minimal effect on pulmonary arterial pressure in a patient with PH secondary to pulmonary emphysema. This case highlights the potential utility of AVP to treat low systemic vascular resistance hypotension in patients with PH secondary to lung diseases.

## Background

Surgery and anesthesia pose a significant risk for patients with pulmonary hypertension (PH) [1–3]; therefore, perioperative management of these patients presents a major challenge to physicians. Several studies in patients undergoing cardiac surgery have demonstrated that arginine vasopressin (AVP) can effectively ameliorate systemic hypotension without increasing pulmonary vascular resistance [4–6]. These results support the administration of AVP for the treatment of hypotension concomitant with PH in the cardiac surgery setting. However, to our knowledge, no previous studies have reported the effect of AVP on the systemic and pulmonary circulation of patients with PH secondary to lung diseases. In this report, we present the hemodynamic responses to bolus administrations of AVP and noradrenaline (NAD) in a patient with PH secondary to pulmonary emphysema. The patient showed low systemic vascular resistance hypotension during off-pump single-lung transplantation. We discuss the challenge of managing intraoperative hypotension in a patient with PH, with focus on the choice of vasopressor in this setting.

## Case presentation

A 61-year-old man (174 cm, 56 kg) with pulmonary emphysema and secondary PH underwent off-pump, single-right-lung transplantation from a brain-dead donor. His preoperative status was Hugh–Jones class IV (unable to walk more than about 50 m without rest), and arterial blood gas analysis at admission showed a PaO_2_ of 74.9 mm Hg and a PaCO_2_ of 35.3 mm Hg at an inhalation rate of 1 L/min O_2_. A preoperative transthoracic echocardiogram showed normal left ventricular systolic function (ejection fraction, 60%) and PH with an estimated systolic pulmonary arterial pressure (PAP) of 66 mm Hg. Symptoms of right heart failure, such as lower-limb edema, were not present. Written informed consent was obtained from the patient for the publication of this case report.

**Fig. 1:**
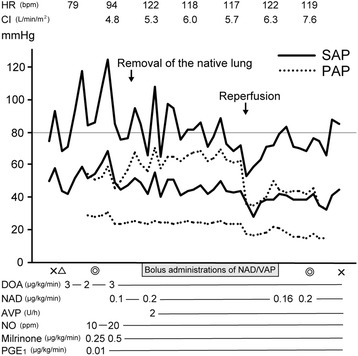
Anesthetic chart. Double circle marks represent start/end of surgery; cross marks represent start/end of anesthesia; a triangle mark represents endtracheal intubation

The anesthetic chart, with a focus on hemodynamic parameters and vasoactive drugs, is presented in Fig. 1. Upon entering the operating room, standard monitoring devices, including an electrocardiograph and a pulse oximeter, were attached to the patient, and a peripheral venous catheter was inserted. In addition, an arterial catheter was inserted into the right radial artery. Anesthesia was induced with midazolam (2 mg), propofol (60 mg), and fentanyl (250 μg) and maintained with sevoflurane (0.8–1%) and remifentanil (0.05–1 μg/kg/min). A pulmonary arterial catheter was inserted after induction of anesthesia and intubation. Initial pulmonary artery catheter readings revealed moderate PH, as indicated by a systemic arterial pressure (SAP) and PAP of 87/52 and 52/28 mm Hg, respectively. Inhalation of nitric oxide (10–20 ppm) along with continuous infusion of milrinone (0.25–0.5 μg/kg/min) and prostaglandin E1 (0.01 μg/kg/min) was initiated, but neither SAP nor PAP changed significantly in response to the administration of these drugs. After the right pulmonary artery was clamped, systolic PAP increased to 60–70 mm Hg.

The patient exhibited low systemic vascular resistance hypotension (systemic vascular resistance, 280–520 dyne s cm^−5^; systolic SAP, 70–90 mm Hg) during the surgery. Therefore, continuous infusion of dopamine (2–3 μg/kg/min), NAD (0.1–0.2 μg/kg/min), and AVP (2 U/h) was administered to maintain SAP. In addition, bolus administrations of NAD (10 or 20 μg, administered 15 times) or AVP (0.5 U, administered twice) were used from the time of clamping of the pulmonary artery until initiation of reperfusion. The intervals between bolus administrations of NAD and AVP ranged from 3 to 12 min. The patient’s cardiac index remained high (4.1–7.6 L/min/m^2^) throughout the operation.

**Fig. 2:**
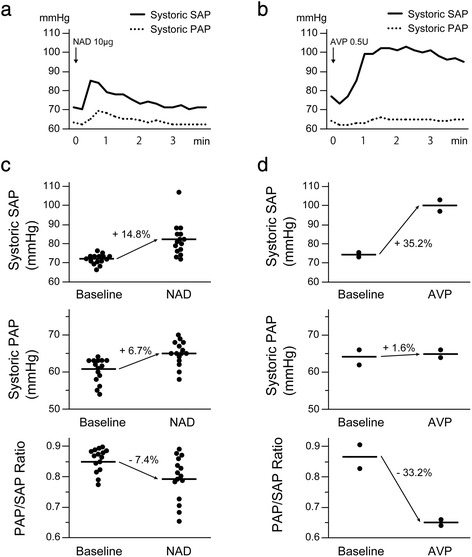
Hemodynamic responses to bolus doses of NAD and AVP. **a**, **b** Representative responses of blood pressure to bolus doses of NAD (**a**) and AVP (**b**). **c**, **d** Changes in blood pressure before and after bolus doses of NAD (**c**) and AVP (**d**). Blood pressure (PAP or SAP) just before the administration of NAD or AVP was considered the baseline level. The peak blood pressures within 3 min after the administration of NAD or AVP were obtained. *Horizontal lines* represent mean values. *NAD* noradrenaline, *AVP* arginine vasopressin, *PAP* pulmonary arterial pressure, *SAP* systemic arterial pressure

Figure 2 shows the patient’s hemodynamic responses to bolus doses of NAD and AVP administered from the time of clamping of the pulmonary artery until reperfusion. The bolus administration of NAD increased systolic SAP by 14.8% relative to that immediately before administration; in addition, it increased systolic PAP by 6.7% relative to pretreatment values (Fig. 2a, c). The bolus administration of AVP also increased systolic SAP by 35.2%; however, it affected PAP only minimally, resulting in a significant decrease in the PAP/SAP ratio from 0.87 to 0.65 (Fig. 2b, d).

Systolic PAP decreased with reperfusion and remained in the range of 40–50 mm Hg thereafter. Systolic SAP also decreased after reperfusion and several bolus doses of NAD were required, although this stabilized within about 40 min. The transplanted lung had adequate perfusion and oxygenation. The operating time was 358 min and the total volume of blood loss was 613 mL; no blood transfusion was required. The postoperative course was largely uneventful, except for a tracheostomy, which was performed on postoperative day 2, owing to pulmonary edema in the transplanted lung. The patient was weaned from the ventilator on postoperative day 8 and from inotropic support on postoperative day 10. The patient was discharged from the hospital 10 weeks after surgery.

### Discussion

Here, we present a case of off-pump single-lung transplantation for pulmonary emphysema concomitant with secondary PH, in which the bolus administration of AVP effectively increased SAP with a minimal effect on PAP.

Maintaining adequate SAP is especially important in patients with PH, because systemic hypotension decreases coronary perfusion pressure, thereby causing myocardial ischemia and right heart failure [7, 8]. The ideal vasopressor agent for patients with PH should increase systemic vascular resistance with a minimal effect on pulmonary vascular resistance and improve the contractility of the right ventricle; however, most vasopressors used in clinical anesthesia and intensive care for the augmentation of systemic vascular resistance will also increase pulmonary vascular resistance [9]. For example, sympathomimetic vasopressors, such as NAD, exert their systemic vasopressor effects through α1-adrenoceptor agonism, which also causes pulmonary vasoconstriction and may potentially impair right ventricular function [10]. In fact, noradrenaline caused elevation of PAP in our patient, although to a lesser degree than that of SAP.

AVP is a nonsympathomimetic vasopressor, which causes vasoconstriction via activation of the V_1_ vasopressin receptor [11]. In vitro and in vivo studies have demonstrated that AVP produces systemic vasoconstriction with relatively fewer effects on the pulmonary circulation; such combined effects produce a desirable decrease in the pulmonary vascular resistance/systemic vascular resistance ratio [12–14]. This AVP-mediated selective vasoconstriction of the systemic vessels has also been shown in several clinical studies in patients undergoing cardiac surgery [4–6]. However, the pathological mechanisms of PH in patients who undergo cardiac surgery are different from those in patients with PH secondary to lung diseases. PH in cardiac surgery patients is commonly caused by elevated pulmonary venous pressure, whereas that secondary to lung diseases is caused by alterations in lung structure or function [15]. To date, data on the effect of AVP on systemic and pulmonary circulation in patients with PH secondary to lung diseases are lacking. In our patient, the bolus administration of AVP selectively increased SAP, which seems to reflect selective vasoconstriction of the systemic vessels. This phenomenon suggests that patients with PH secondary to lung diseases also benefit from the administration of AVP for the treatment of systemic hypotension.

The data presented in this report should be interpreted cautiously. Although the authors analyzed the hemodynamic responses to bolus administrations of AVP and NAD, continuous infusion of these drugs might have different effects. In addition, several drugs, including AVP and NAD, that have influence on systemic and pulmonary vascular resistance were continuously administered during the surgery. Although the administration flow rate of these drugs was constant in most of the period from the time of clamping of the pulmonary artery until reperfusion, continuous infusion of these drugs might have affected the observed hemodynamic responses.

Although AVP is not indicated for treatment of hypotension in Japan, AVP was used without the approval of the Ethics Committee in this case because it was an emergent situation in which hypotension persisted despite the administration of NAD and we did not have time to wait for Ethics Committee approval. However, AVP use in vasodilatory shock has been shown to be safe in many studies [16] and was approved in 2014 by the Food and Drug Administration [17]. We consider that AVP use in this case was not ethically problematic.

The potential risks associated with the administration of AVP should be carefully considered. AVP may cause myocardial dysfunction probably owing to direct myocardial effects, including coronary vasoconstriction [18]. Cardiac function should be closely monitored using a pulmonary artery catheter or transesophageal echocardiography during the administration of AVP.

## Conclusions

The authors observed a 35.2% increase in SAP with a minimal change in PAP, resulting in a significant decrease in the PAP/SAP ratio after the bolus administration of AVP in a patient with PH secondary to pulmonary emphysema. This case highlights the potential utility of AVP to treat low systemic vascular resistance hypotension in patients with PH secondary to lung diseases.

## Abbreviations

AVP: Arginine vasopressin
NAD: Noradrenaline
PAP: Pulmonary arterial pressure
PH: Pulmonary hypertension
SAP: Systemic arterial pressure
